# Heterogenous mismatch-repair status in colorectal cancer

**DOI:** 10.1186/1746-1596-9-126

**Published:** 2014-06-26

**Authors:** Patrick Joost, Nynke Veurink, Susanne Holck, Louise Klarskov, Anders Bojesen, Maria Harbo, Bo Baldetorp, Eva Rambech, Mef Nilbert

**Affiliations:** 1 Department of Oncology and Pathology, Institute of Clinical Sciences, Lund University, SE-22381, Lund, Sweden; 2 Department of Pathology and Clinical Research Centre, University Hospital of Copenhagen, DK-2650, Hvidore, Denmark; 3 Department of Pathology, Herlev Hospital, DK-2730, Herlev, Denmark; 4 Department of Clinical Genetics, Vejle Hospital, DK-7100, Vejle, Denmark

**Keywords:** Mismatch repair, immunohistochemistry, heterogeneity, MLH1, MSH2, MSH6, PMS2

## Abstract

**Abstract:**

**Background**

Immunohistochemical staining for mismatch repair proteins is efficient and widely used to identify mismatch repair defective tumors. The tumors typically show uniform and widespread loss of MMR protein staining. We identified and characterized colorectal cancers with alternative, heterogenous mismatch repair protein staining in order to delineate expression patterns and underlying mechanisms.

**Methods**

Heterogenous staining patterns that affected at least one of the mismatch repair proteins MLH1, PMS2, MSH2 and MSH6 were identified in 14 colorectal cancers. Based on alternative expression patterns macro-dissected and micro-dissected tumor areas were separately analyzed for microsatellite instability and *MLH1* promoter methylation.

**Results**

Heterogenous retained/lost mismatch repair protein expression could be classified as intraglandular (within or in-between glandular formations), clonal (in whole glands or groups of glands) and compartmental (in larger tumor areas/compartments or in between different tumor blocks). These patterns coexisted in 9/14 tumors and in the majority of the tumors correlated with differences in microsatellite instability/MLH1 methylation status.

**Conclusions**

Heterogenous mismatch repair status can be demonstrated in colorectal cancer. Though rare, attention to this phenomenon is recommended since it corresponds to differences in mismatch repair status that are relevant for correct classification.

**Virtual Slides:**

The virtual slide(s) for this article can be found here: http://www.diagnosticpathology.diagnomx.eu/vs/1771940323126788

## Background

Mismatch repair (MMR) defects characterize 2-4% of colorectal cancers linked to Lynch syndrome and 15% of sporadic colorectal cancers caused by epigenetic *MLH1* promoter methylation. Various strategies can be used to preselect colorectal cancers for MMR protein testing, e.g. clinical guidelines for hereditary cancer, MMR prediction models that combine clinical and pathological information and potentially novel biomarker-based strategies [1-5].

Universal assessment of immunohistochemical MMR staining is increasingly applied in colorectal cancer diagnostics in order to identify cases suspected of Lynch syndrome for further molecular diagnostics and to obtain treatment-predictive information linked to somatic methylation of *MLH1*[6].

The monoclonal antibodies used for immunohistochemical MMR protein staining generally result in stable and consistent staining patterns with retained staining or loss of staining. Functional interaction between the MLH1/PMS2 and the MSH2/MSH6 proteins implies that the expression pattern of the heterodimerizing protein partner may be used to direct mutation analysis. Aberrant MMR function typically leads to complete loss of nuclear staining in the tumor cells, particularly when linked to *MLH1* promoter hypermethylation that leads to complete gene silencing [7]. In Lynch syndrome, the multitude of disease-predisposing mutations may have variable effects on epitope expression, from complete loss to weak or retained expression for one or both heterodimerizing proteins [8,9]. Variable epitope expression may also result in alternative expression patterns, e.g. cytoplasmic staining and perinuclear staining, which are typically present throughout the tumor [10]. MMR protein immunostainings are generally stable and relatively easy to interpret; though challenges and pitfalls have been reported with false positive as well as false negative interpretations [10-12]. Most commonly, these observations relate to technical artifacts caused by suboptimal fixation or paraffin-embedding, necrotic areas, sample storage, antibody specificity, clone selection or staining conditions [13,14]. Also, neoadjuvant chemotherapy and radiotherapy may influence the results with a particular effect on MSH2/MSH6 staining [15,16]. Heterogenous expression patterns with retained staining in the adenomatous part and loss of staining in a smaller, invasive part of the tumor have been reported but their relevance is uncertain [17]. We systematically collected colorectal cancers with heterogenous MMR protein staining patterns for detailed analysis with correlations e.g. to MSI status and *MLH1* promoter methylation.

## Methods

### Materials

Colorectal cancers with heterogenous MMR protein expression were identified during evaluations at the Departments of Pathology, Helsingborg Hospital, Sweden and Hvidore Hospital, Denmark. Following the first observation of heterogenous MMR protein staining in 2007, two gastrointestinal pathologists (PJ and SH) collected all such cases identified at these two institutions during 5 years. In total, 14 colorectal cancers with heterogenous MMR protein expression were identified for in-depth analysis (Table 1). The materials consisted of resection specimens from 12 colon cancers and 2 rectal cancers. None of the patients had received neoadjuvant radiotherapy or chemotherapy. All cases were histologically re-evaluated by one pathologist (P.J.). Tumor stage was determined according to the American Joint Cancer Committee/Union Internationale Contre le Cancer (AJCC/UICC) staging system and the grade according to the WHO system. Mucinous cancers were considered poorly differentiated. A tumor was classified as mucinous cancer if more than 50% of the tumor area showed such differentiation [18]. Tumors with mucinous components that encompassed &50% of the area were classified as having a mucinous component, though not fulfilling the criteria for mucinous tumors [19]. Mucinous cancer was observed in 6 cases. MMR gene mutation testing had been performed in 8 cases, 4 of which carried disease-predisposing mutations. Ethical approval for the study was granted from the ethical committees at Lund University, Sweden and at the Capital Region, Copenhagen.

**Table 1:** Summary of clinical and pathological data

**Case**	**Sex/Age**	**Tumor location**	**Histologic type**	**Differentiation**	**pTNM**	**Stage**	**MMR gene mutation**
1	F/57	Cecum	Adca	Moderate	T2N0MX	I	Not tested
2	F/33	Cecum	Mucinous adca	Poor	T2N0MX	I	*MSH2*
4	M/41	Rectum	Adca	Moderate	T3N0MX	II	*MSH6*
5	F/63	Transverse colon	Mucinous adca	Moderate	T4N1MX	III	Not tested
6	F/82	Rectum	Mucinous adca	Moderate	T2N0MX	I	Not identified
8	M/85	Cecum	Adca	Poor	T4N0MX	II	Not identified
9	M/71	Ascending colon	Mucinous adca	Poor	T3N0MX	II	Not tested
10	F/66	Ascending colon	Mucinous adca	Poor	T3N0MX	II	Not identified
11	F/57	Descending colon	Adca	Moderate	T3N0MX	II	*MSH6*
12	F/55	Ascending colon	Adca	Poor	T3N0MX	II	*MLH1*
14	M/82	Ascending colon	Adca	Moderate	T3N0MX	II	Not tested
15	M/83	Ascending colon	Adca	Moderate	T3N0MX	II	Not tested
16	M/74	Transverse colon	Mucinous adca	Poor	T3N1MX	III	Not tested
17	F/74	Cecum	Adca	Poor	T3N1MX	III	Not identified

*F*: female; *M*: Male; Adca: adenocarcinoma; *MMR*: mismatch repair.

### MMR protein immunostaining

Sections from all tumor blocks (n = 4-11) from the 14 cases were subjected to independent MMR protein staining using alternative MMR protein antibodies from other manufacturers (Table 2). Fresh 4-μm sections from formalin-fixed, paraffin-embedded tumors were mounted on Dako REAL™ Capillary gap microscope slides (Dako, Glostrup, Denmark). The slides were dried overnight at room temperature and thereafter at 60°C for 1–2 hours. The tissue was deparaffinized in xylene for two times 5 min, followed by 5 min each in 99.5% and 95% ethanol and 5 min in distilled water. Heat-induced epitope retrieval was achieved by pressure boiler-treatment in ethylene diamine tetraacetic acid (EDTA)-Tris buffer (1:10 mM, pH 9.0) for 20 min. Hereafter, the slides were cooled for 20 min and rinsed in distilled water. Immunostaining was performed using the Dako Autostainer and the EnVision™ visualization method (Dako, Glostrup, Denmark). Endogenous peroxidase activity was blocked for 5 min and primary mouse monoclonal IgG antibodies were used (Table 2). Following primary antibody incubation, the slides were incubated with EnVision™/horseradish peroxidase (HRP) rabbit/mouse (Dako) and stained using the EnVision™ Detection System peroxidase/DAB rabbit/mouse (Dako). The immunohistochemical stainings were classified as retained, lost or reduced, i.e. a weaker than expected staining in the tumor cells compared to the stromal cells. The areas of the respective expression patterns were estimated in each block end expressed in percentage.

**Table 2:** Information on the MMR protein antibodies used

**MMR protein**	**Lab**	**Supplier**	**Clone**	**Dilution**	**Immunogen/epitope**
MLH1	L	BD Pharmingen	G168-15	1:100	Full-length
	C	DAKO	ES05	RTU	Recombinant protein, 210 aa
	L	DAKO	ES05	1:100	Recombinant protein, 210 aa
PMS2	L	BD Pharmingen	A16-4	1:300	aa 431–862, C-terminal
	H	Ventana (Cell Marque)	EPR3947	RTU	100 aa, C-terminal
	C	Epitomics	EPR3947	1:50	100 aa, C-terminal
MSH2	L	Calbiochem	FE11	1:100	C-terminal
	H	Ventana (Cell Marque)	G219-1129	RTU	Full-length
	C	Novocastra	25D12	1:50	Full-length
MSH6	C	Epitomics	EP49	1:100	Synthetic peptide, N-terminal
	L	Epitomics	EP3945	1:100	Synthetic peptide, N-terminal
	L	BD Transduction Lab.	44	1:500	Synthectic peptide aa 225-333

*MMR*: mismatch repair; *L*: Lund; H Helsingborg; *C*: Copenhagen; *RTU*: ready to use.

### Microsatellite instability analysis

To assess the impact from heterogenous MMR protein stainings on MMR protein function, the tumors were subjected analysis of microsatellite instability (MSI). Depending on the extent of the area involved, microdissection or macrodissction was used to obtain material from areas with the respective expression patterns (Table 3). Laser capture micro-dissection was performed using Polyethylene Teraphthalate (PET)-membrane FrameSlides (Carl Zeiss MicroImaging, Germany). In tumors where larger areas showed variable expression patterns, macro-dissection was performed. Tissue from 2–6 tumor areas with the respective MMR protein stained patterns were collected from 10-μm tissue sections. DNA extraction was performed using QIAamp® DNA micro kit (Qiagen) for laser micro-dissected tissues and using the QIAcube machine (Qiagen) or the QIAamp® DNA FFPE tissue kit (Qiagen) for macro-dissected tissues or whole tumor sections. MSI analyses were performed using the MSI Analysis System, Version 1.2 (Promega, Madison, WI), PCR products were size separated on a 3130*xl* Genetic Analyzer (Applied Biosystems, Foster City, CA). The results were evaluated using the GeneMapper® software Version 4.0 (Applied Biosystems, Foster City, CA). The analysis included the 5 mononucleotide markers BAT-25, BAT-26, NR-21, NR-24, and MONO-27 (Promega, MSI Analysis System, Version 1.2, Madison, WI). Tumors with instability for 1 marker were classified as MSI low, tumors with instability for ≥2 markers were classified as MSI-high (MSI-H), and tumors with stability for all markers were classified as microsatellite stable (MSS).

**Table 3:** Summary of MMR heterogeneity

**Case**	**Block no**.	**Hetero**-**geneity**	**Pattern**	**Involved area (%)**	**MMR protein immunostaining**				**Fraction of MSI in markers**	**MLH1 promoter methylation**
					**MLH1**	**PMS2**	**MSH2**	**MSH6**		
1	1A¤/*	+	CL	15	+/−	+/−	+	+	0/5 and 4/5	+/−
	1B	+	CL	5	-	-	+/R	+/−		
	1C	-	H	0	-	-	+	+		
	1D	-	H	0	-	-	+	+		
	1E¤	+	CL, IG	70 / 10	+/−	+/−	+/R	+/−	0/3 vs 4/5	
2	24	-	H	0	+	+	+	+		-
	25*	-	H	0	+	+	+	+	0/5	
	26	-	H	0	+	+	+	+		
	27*	-	H	0	+	+	+	+	0/5	
	28	+	CL, IG	15	+/−	+/−	+	+		
	29*	+	CL, IG	40	+/−	+/−	+	+	5/5	
	30	+	IG	10	+/−	+/−	+	+		
	31	+	IG	5	+/−	+/−	+	+		
	32	+	IG	5	+/−	+/−	+	+		
4	2	+	IG	97	+	+	+/−	R/-		NE
	3¤/*	+	CL, IG	95	+	+	+/−	R/-	3/3 vs 3/5	
	4	+	CL, IG	80	+	+	+/−	R/-		
	5*	+	IG	95	+	+	+/−	R/-	5/5	
5	20*	+	CL, IG	50 / 100	+	+/−	-	R/-	5/5	-
	21	+	IG	100	+	+	-	R/-		
	23	+	IG	100	+	+	-	R/-		
	24	+	IG	100	+	+	-	R/-		
	25	+	COM, IG	100	+	-	-	R/-		
	26	+	COM, IG	100	+	-	-	R/-		
	27	+	COM IG	100	+	-	-	R/-		
	28	+	COM, IG	100	+	-	-	R/-		
	29	+	COM, IG	100	+	-	-	R/-		
	30	+	COM, IG	100	+	-	-	R/-		
	31	+	COM, IG	100	+	-	-	R/-		
6	5¤	+	IG	100	+	R/-	+	+	0/5 vs 0/4	NE
	6*	+	IG	100	+	R/-	+	+	0/5	
	7*	-	H	0	+	+	+	+	0/5	
	8*	+	IG	100	+	R/-	+	+	0/5	
	9*	+	IG	95	+	R/-	+	+	0/5	
	10	+	IG	95	+	R/-	+	+		
	11*	+	IG	100	+	R/-	+	+	0/5	
8	5*	-	H	0	-	-	+	+	5/5	+
	6	+	CL, IG	60	-	-	+	R/-		
	7	+	CL	20	-	-	+	+/−		
	8	-	H	0	-	-	+	+		
	9	-	H	0	-	-	+	+		
	10	+	CL, IG	20	-	-	+/R	+/−		
	11	-	H	0	-	-	+	+		
	12	+	CL	20	-	-	+/−	+/−		
9	16	-	H	0	+	+	+	+		+
	19	-	H	0	+	+	+	+		
	20*	-	H	0	+	+	+	+	0/5	
	21*	-	COM	100	-	-	+	+	5/5	
	22	-	H	0	+	+	+	+		
	40	-	H	0	+	+	+	+		
	44	-	H	0	+	+	+	+		
10	6	+	CL	50	-	-	+/−	+/−		+
	7	-	H	0	-	-	+	+		
	8	-	H	0	-	-	+	+		
	9*	-	H	0	-	-	+	+	5/5	
	10	-	H	0	-	-	+	+		
	11	-	H	0	-	-	+	+		
11	3	-	H	0	-	-	+	+		+
	4	+	CL, IG	5	-	-	+/−	+/−		
	5	-	H	0	-	-	+	+		
	6*	-	H	0	-	-	+	+	5/5	
	7	-	H	0	-	-	+	+		
	8*	+	CL, IG	30	-	-	+/R	+/−	5/5	
12	3*	+	CL	5	-	-	+/−	+/−	5/5	+
	4	+	CL, IG	90	-	-	+/−	+/−		
	5	+	CL, IG	80	-	-	+/−	+/−		
	6	-	H	100	-	-	-	-		
	7	+	CL	60	-	-	+/−	+/−		
	8	+	CL, IG	90	-	-	+/−	+/−		
	9*	+	CL, IG	60	-	-	+/−	+/−	5/5	
14	1A*	-	H	0	-	-	+	+	5/5	+
	1B*	+	CL	5	-	-	+/R	+/−	5/5	
	1C*	+	CL, IG	30	-	-	R/-	+/−	5/5	
	1D*	+	CL	40	-	-	+/R	+/−	5/5	
	1E*	+	CL, IG	40	-	-	R/-	+/−	5/5	
15	4A*	+	IG	100	+	+	+/R	R/-	5/5	NE
	4B*	+	IG	100	+	+	+	R/-	5/5	
	4C	+	IG	100	+	+	+/R	R/-		
	4D	+	IG	100	+	+	+	R/-		
	4E	+	IG	100	+	+	+/R	R/-		
16	6A	+	IG	15	-	-	R	+/−		+
	6B*	+	CL	3	-	-	+/R	+/−	5/5	
	6C	+	CL	10	-	-	+/R	+/−		
	6D	+	IG	5	-	-	R	+/−		
	6E	+	IG	5	-	-	+/R	+/−		
17	18*	+	CL	55	-	-	+/−	+/−	5/5	+
	19	-	H	0	-	-	+	+		
	20	+	CL	8	-	-	+/−	+/−		
	21	-	H	0	-	-	+	+		
	22	-	H	0	-	-	+	+		

microdissection; *: macrodissection; *CL*: clonal, *IG*: intraglandular; *COM*: compartmental; *H*: homogenous; *R*: reduced staining; +/-: heterogenous staining; *MMR*: mismatch repair; *MSI*: microsatellite instability; *NE*: not evaluated.

### Methylation-specific PCR analysis

Extracted DNA was treated with bisulfite using the EZ DNA Methylation-Lightning™ Kit (Zymo Research, CA, USA) according to the manufacturer’s instructions. MLH1 promoter methylation status was analyzed by means of a fluorescence-based, real-time methylation-specific PCR assay, as described previously [20]. Two sets of primers and probes, designed specifically for bisulfite-converted DNA, were used: MLH1-M2B for the methylation-specific reaction [21] and ALU-C4 for the methylation independent control reaction used to measure the amount of bisulfite-converted input DNA [22]. Amplification was performed on QuantStudio™ 12 K Flex Real-Time PCR System (Life Technologies). Samples were run in duplicate, including positive and negative controls.

### Flow cytometry

Flow cytometric DNA analysis was performed as previously described [23,24]. The separated cells were then treated with ribonuclease (Sigma-Aldrich, Stockholm, Sweden), incubated with trypsin for 48 h (Merck, Darmstadt, Germany), and stained with propidium iodide (Sigma-Aldrich, Stockholm). Flow cytometric DNA analysis was performed in a FACS Caliber (Becton, Dickinson, BD Biosciences, USA). Up to 20,000 nuclei were analysed from each sample. The DNA histograms obtained were automatically processed using Modfit LT 3.3™ software. The DNA index (DI) was calculated as the ratio of the respective modal channel values of the non-diploid and the diploid G0/G1 peaks. The S-phase fraction (Spf) was estimated assuming that the S-phase compartment constituted a rectangular distribution between the modal values of the G0/G1 and G2 peaks.

## Results

**Figure 1:**
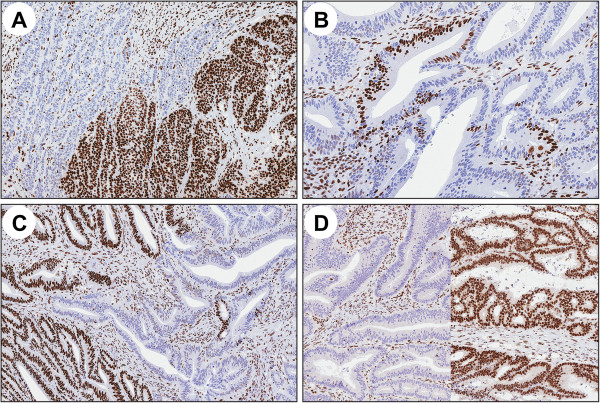
**Examples of the different MMR protein staining patterns. A)** clonal loss, **B)** intraglandular loss, **C)** co-existence of clonal and intraglandular loss and **D)** compartmental loss with different patterns in two separate tumor blocks.

Immunohistochemical staining using alternative MMR protein antibodies confirmed heterogenous MMR protein expression in all 14 tumors. Heterogenous expression affected MLH1/PMS2 in 3 tumors, PMS2 in 2 tumors, MSH2/MSH6 in 10 tumors (of which two also expressed heterogeneity for MLH1/PMS2) and MSH6 only in 1 tumor (in which one block also expressed heterogeneity for PMS2). Areas with alternative expression patterns were well demarcated and appeared in three distinct patterns: “intraglandular” (retained/lost staining within or in between glandular formations), “clonal” (retained/lost staining in whole glands or groups of glands) and “compartmental” (retained/lost staining in larger tumor areas/compartments leading to retained/lost staining in between different tumor blocks) (Figure 1, Table 3). Various heterogenous expression patterns co-existed in 9/14 tumors, most commonly as intraglandular and clonal heterogeneity (figure 1c). The heterogenous staining patterns were present in 3-100% of the examined tumor area. In 4/14 cases, all tumor blocks showed heterogeneity, whereas the remaining tumors showed heterogeneity in a variable fraction of the tumor blocks (Table 3).

**Figure 2:**
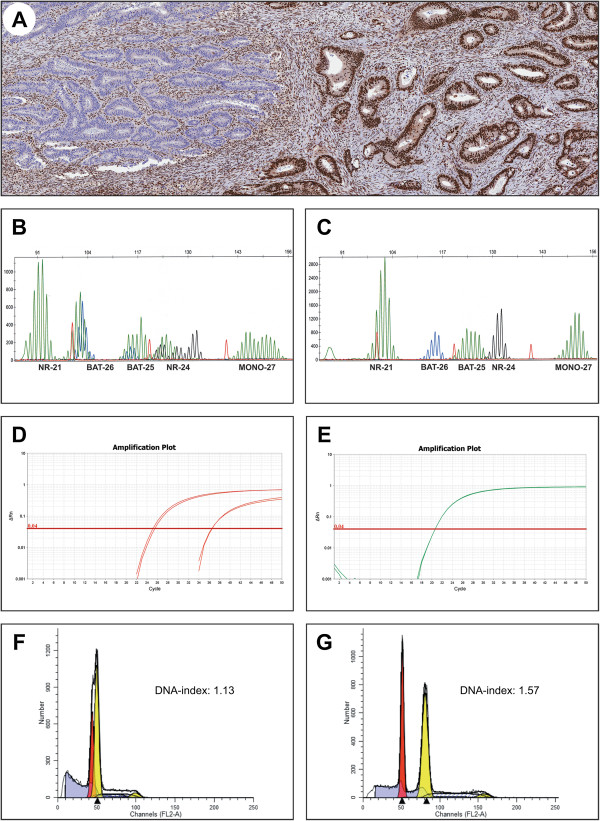
**An adenocarcinoma (case 1) with 4 different expression patterns and various combinations of heterogeneity**, **loss of MLH1/PMS2 and heterogeneity/retained expression for MSH2/MSH6. A)** clonal loss of MLH1 staining. **B)** MSI corresponding to loss of staining, **C)** MSS corresponding to retained MMR protein staining. Methylation analysis revealed **D)** presence and **E)** absence, respectively, of *MLH1* promoter methylation, which verifies clonal *MLH1* methylation status. Flow cytometric analysis showing different DNA indices, i.e. **F)** 1.13 in the MSI area and **G)** 1.57 in the MSS area.

MSI was demonstrated in 13/14 tumors. Intra-tumor differences in MMR status, i.e.MSI *versus* MSS, in line with MMR protein staining expression was observed in 3 tumors (Table 3; case 1, 2 and 9). Non-consistent, homogenous MSI status in tumors with heterogenous MMR protein expression was observed in 2 cases (Table 3; cases 4 and 6). *MLH1* promoter methylation was demonstrated in all 7 cases with complete (non-heterogenic) loss of MLH1/PMS2. In 2 cases (Table 3; cases 1 and 9) heterogenous MMR protein staining for MLH1/PMS2 correlated with heterogenous *MLH1* promoter methylation, i.e. tumor areas with retained MLH1 expression did not show MLH1 methylation, whereas areas with loss of MLH1 expression showed MLH1 methylation. Concordant immunostaining and methylation status suggest functional intratumoral heterogeneity (Figure 2). DNA flow cytometric analysis was performed in one tumor (case 1) and demonstrated differences in DNA content within the heterogenous areas, which had DNA indices of 1.13 and 1.57, respectively (Figure 2).

## Discussion

Heterogeneous MMR protein expression is a rare phenomenon, but corresponds to differences in MMR status within the tumor and is therefore important to recognize to prevent false-positive or false-negative evaluations. We identified three distinct patterns of heterogeneity, i.e. intraglandular, clonal and compartmental heterogenous MMR protein expression. The different patterns co-existed within the same tumor and the extent of the tumor involved varied. In-depth analysis suggests that multiple causes may apply, e.g. variable epitope expression, expression related to variable differentiation, second hit mutations or methylation in selected tumor clones and possibly influence from factors linked to the tumor microenvironment such as hypoxia and oxidative stress [25].

Intraglandular and/or clonal heterogeneity throughout the tumor, which may be caused by variable epitope expression, was identified in 4 tumors (cases 4, 5, 15 and 16, Table 3). Homogenous loss of MLH1/PMS2 and heterogenous expression of MSH2/MSH6 was identified in 7 tumors that were consistently MSI and showed *MLH1* promoter methylation (Table 3). This expression pattern has previously been observed and may relate either to a germline *MSH2*/*MSH6* mutation that allows for partial epitope binding in the presence of somatic *MLH1* methylation or to secondary *MSH2*/*MSH6* inactivation [25-27]. Heterogenous MLH1 and/or PMS2 expression, suggestive of variable *MLH1* methylation/second hit mutations was observed in 2 tumors (case 2 and 6, Table 3). Case 1 showed a more complex pattern of MMR protein expression and intra-tumor differences in MSI, *MLH1* promoter methylation and DNA content, suggestive of a tumor composed of two distinct clones (case 1, Figure 2).

**Figure 3:**
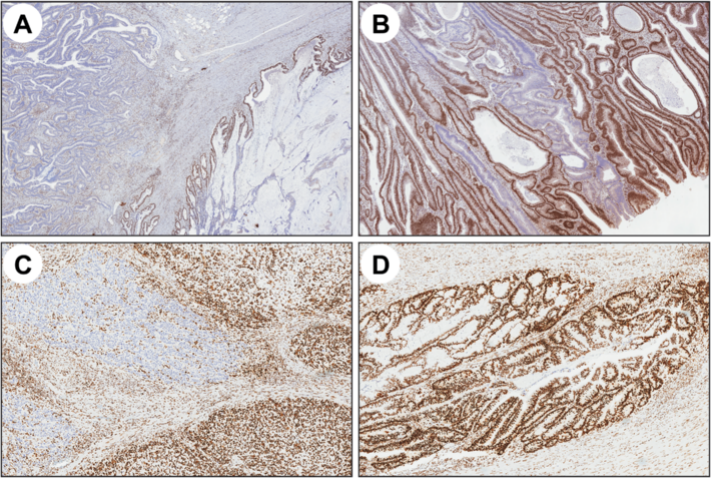
**Variable MMR protein expression in relation to tumor differentiation. A)** case 5 with retained expression for PMS2 in a mucinous tumor component and loss of PMS2 expression in a non-mucinous component. **B)** case 2 with clonal and intraglandular heterogeneity for MLH1 in the adenomatous component of the tumor, whereas the remaining tumors showed retained expression for MLH1. **C**-**D)** case 17 with clonal heterogeneity for MLH6 in a poorly differentiated tumor component and homogenous expression in a well-differentiated tumor component.

Variable MMR status did in some tumors correspond to variable differentiation, e.g. mucinous areas (cases 5 and 15), poor differentiation (cases 16 and 17) or adenomatous components (case 2, Figure 3). Correlation between MSI status and expression also of other molecular markers has been described [28,29]. Different, through homogenous, MMR protein expression patterns in distinct tumor compartments were observed in a mucinous adenocarcinoma (case 9) with loss of MLH1/PMS2 expression, MSI and *MLH1* methylation in 1/7 tumor blocks that corresponded to an adenomatous tumor component (Figure 1d). Sample mix was excluded through histologic review and penta-D marker fragment analysis (data not shown). Homogenous loss/reduced staining of MSH2/MSH6 throughout the tumor and additional loss of PMS2 in 7/10 tumor blocks was observed in a mucinous adenocarcinoma (case 5). This case most likely reflects how the mucinous tumor component progressed in another line than the non-mucinous tumor component. Though compartmental loss of MMR protein expression is rare, this observation motivates thorough evaluation of different tumor compartments, particularly when areas with variable expression are identified.

Limitations to our study include analysis based on surgical specimens though biopsy material may produce stainings of better technical quality [30-33]. At the same time use of biopsy material implies analysis of a restricted tumor area that may not capture areas with alternative expression. Also, information on MMR gene mutation status was not available in all cases. The 4 tumors from Lynch syndrome mutation carriers, however, expressed heterogeneity in between different tumor blocks, which showed homogenous as well as heterogenous loss in clonal and intraglandular patterns. Recognition of so-called “patchy” MMR protein staining has been reported and were also considered herein (Table 3). This phenomenon differs from the heterogenous staining patterns described herein in that it primarily relates to MSH6 stainings, neoadjuvant treatment [15,16] or represents a weak or cytoplasmic staining rather than the distinct and well-demarcated areas of retained staining and loss of staining described herein.

## Conclusions

Our study verifies heterogenous MMR status in a subset of colorectal cancer. Heterogenous MMR protein expression appears in three major forms, which frequently co-exist and correlate to differences in MMR status. We suggest that variable MMR protein staining patterns should be considered and when observed linked to extended analysis in order to ensure correct classification of MMR status.

## Abbreviations

MMR: Mismatch repair; MSI: Microsatellite instability; MSI-H: MSI-high; MSS: Microsatellite stable.

## Competing interests

The author’s declare that they have no competing interests.

## Authors' contributions

PJ: corresponding author, study design, review of pathology samples, data analysis and manuscript writing, NV: data collection, macrodissection, MSI analysis, SH: identification of relevant cases, histopathologic review, LK: identification of relevant cases, histopathologic review, AB/MH: MLH1 methylation analysis, BB: DNA ploidy analysis, ER: sample collection, MMR protein immunostaining, MN: study design, data analysis, MMR evaluation, manuscript review. All authors read and approved the final manuscript.

## References

[B1] Kastrinos F Balmana J Syngal S Prediction models in Lynch syndrome Fam Cancer 2013 12 217 228 23553450 10.1007/s10689-013-9632-0 PMC3757552

[B2] Steinke V Holzapfel S Loeffler M Holinski-Feder E Morak M Schackert HK Gorgens H Pox C Royer-Pokora B von Knebel-Doeberitz M Buttner R Propping P Engel C Evaluating the performance of clinical criteria for predicting mismatch repair gene mutations in Lynch syndrome: a comprehensive analysis of 3,671 families Int J Cancer 2014 135 69 77 24493211 10.1002/ijc.28650

[B3] Umar A Boland CR Terdiman JP Syngal S de la Chapelle A Ruschoff J Fishel R Lindor NM Burgart LJ Hamelin R Hamilton SR Hiatt RA Jass J Lindblom A Lynch HT Peltomaki P Ramsey SD Rodriguez-Bigas MA Vasen HF Hawk ET Barrett JC Freedman AN Srivastava S Revised Bethesda Guidelines for hereditary nonpolyposis colorectal cancer (Lynch syndrome) and microsatellite instability J Natl Cancer Inst 2004 96 261 268 14970275 10.1093/jnci/djh034 PMC2933058

[B4] Vasen HF Watson P Mecklin JP Lynch HT New clinical criteria for hereditary nonpolyposis colorectal cancer (HNPCC, Lynch syndrome) proposed by the International Collaborative group on HNPCC Gastroenterology 1999 116 1453 1456 10348829 10.1016/s0016-5085(99)70510-x

[B5] Yatabe J Yatabe MS Ishibashi K Nozawa Y Sanada H Early detection of colon cancer by amino acid profiling using AminoIndex Technology: a case report Diagn Pathol 2013 8 203 24325735 10.1186/1746-1596-8-203 PMC3937238

[B6] Moreira L Balaguer F Lindor N de la Chapelle A Hampel H Aaltonen LA Hopper JL Le Marchand L Gallinger S Newcomb PA Haile R Thibodeau SN Gunawardena S Jenkins MA Buchanan DD Potter JD Baron JA Ahnen DJ Andreu M Ponz de Leon M Rustgi AK Castells A Identification of Lynch syndrome among patients with colorectal cancer JAMA 2012 308 1555 1565 23073952 10.1001/jama.2012.13088 PMC3873721

[B7] Li X Yao X Wang Y Hu F Wang F Jiang L Liu Y Wang D Sun G Zhao Y MLH1 promoter methylation frequency in colorectal cancer patients and related clinicopathological and molecular features PLoS One 2013 8 e59064 23555617 10.1371/journal.pone.0059064 PMC3612054

[B8] Mangold E Pagenstecher C Friedl W Fischer HP Merkelbach-Bruse S Ohlendorf M Friedrichs N Aretz S Buettner R Propping P Mathiak M Tumours from MSH2 mutation carriers show loss of MSH2 expression but many tumours from MLH1 mutation carriers exhibit weak positive MLH1 staining J Pathol 2005 207 385 395 16216036 10.1002/path.1858

[B9] Shia J Klimstra DS Nafa K Offit K Guillem JG Markowitz AJ Gerald WL Ellis NA Value of immunohistochemical detection of DNA mismatch repair proteins in predicting germline mutation in hereditary colorectal neoplasms Am J Surg Pathol 2005 29 96 104 15613860 10.1097/01.pas.0000146009.85309.3b

[B10] Muller A Giuffre G Edmonston TB Mathiak M Roggendorf B Heinmoller E Brodegger T Tuccari G Mangold E Buettner R Ruschoff J Challenges and pitfalls in HNPCC screening by microsatellite analysis and immunohistochemistry J Mol Diagn 2004 6 308 315 15507669 10.1016/S1525-1578(10)60526-0 PMC1867493

[B11] Klarskov L Ladelund S Holck S Roenlund K Lindebjerg J Elebro J Halvarsson B von Salome J Bernstein I Nilbert M Interobserver variability in the evaluation of mismatch repair protein immunostaining Hum Pathol 2010 41 1387 1396 20573374 10.1016/j.humpath.2010.03.003

[B12] Overbeek LI Ligtenberg MJ Willems RW Hermens RP Blokx WA Dubois SV van der Linden H Meijer JW Mlynek-Kersjes ML Hoogerbrugge N Hebeda KM van Krieken JH Interpretation of immunohistochemistry for mismatch repair proteins is only reliable in a specialized setting Am J Surg Pathol 2008 32 1246 1251 18677806 10.1097/pas.0b013e31816401bb

[B13] Engel KB Moore HM Effects of preanalytical variables on the detection of proteins by immunohistochemistry in formalin-fixed, paraffin-embedded tissue Arch Pathol Lab Med 2011 135 537 543 21526952 10.5858/2010-0702-RAIR.1

[B14] Shia J Ellis NA Klimstra DS The utility of immunohistochemical detection of DNA mismatch repair gene proteins Virchows Arch 2004 445 431 441 15455227 10.1007/s00428-004-1090-5

[B15] Bao F Panarelli NC Rennert H Sherr DL Yantiss RK Neoadjuvant therapy induces loss of MSH6 expression in colorectal carcinoma Am J Surg Pathol 2010 34 1798 1804 21107085 10.1097/PAS.0b013e3181f906cc

[B16] Radu OM Nikiforova MN Farkas LM Krasinskas AM Challenging cases encountered in colorectal cancer screening for Lynch syndrome reveal novel findings: nucleolar MSH6 staining and impact of prior chemoradiation therapy Hum Pathol 2011 42 1247 1258 21334712 10.1016/j.humpath.2010.11.016

[B17] Giuffre G Muller A Brodegger T Bocker-Edmonston T Gebert J Kloor M Dietmaier W Kullmann F Buttner R Tuccari G Ruschoff J Microsatellite analysis of hereditary nonpolyposis colorectal cancer-associated colorectal adenomas by laser-assisted microdissection: correlation with mismatch repair protein expression provides new insights in early steps of tumorigenesis J Mol Diagn 2005 7 160 170 15858139 10.1016/S1525-1578(10)60542-9 PMC1867529

[B18] Hamilton SR Bosman FT Boffetta P Ilyas M Morreau H Nakamura SI Quirke P Riboli E Sobin LH Bosman FT, Carneiro F, Hruban RH, Theise ND Carcinoma of the colon and rectum WHO Classification of Tumours of the Digestive System. Volume 3 2010 4 Lyon, France: IARC Press 134 146

[B19] Halvarsson B Anderson H Domanska K Lindmark G Nilbert M Clinicopathologic factors identify sporadic mismatch repair-defective colon cancers Am J Clin Pathol 2008 129 238 244 18208804 10.1309/0PP5GDRTXUDVKAWJ

[B20] Eads CA Danenberg KD Kawakami K Saltz LB Blake C Shibata D Danenberg PV Laird PW MethyLight: a high-throughput assay to measure DNA methylation Nucleic Acids Res 2000 28 E32 10734209 10.1093/nar/28.8.e32 PMC102836

[B21] Fiegl H Gattringer C Widschwendter A Schneitter A Ramoni A Sarlay D Gaugg I Goebel G Muller HM Mueller-Holzner E Marth C Widschwendter M Methylated DNA collected by tampons–a new tool to detect endometrial cancer Cancer Epidemiol Biomarkers Prev 2004 13 882 888 15159323

[B22] Weisenberger DJ Campan M Long TI Kim M Woods C Fiala E Ehrlich M Laird PW Analysis of repetitive element DNA methylation by MethyLight Nucleic Acids Res 2005 33 6823 6836 16326863 10.1093/nar/gki987 PMC1301596

[B23] Baldetorp B Dalberg M Holst U Lindgren G Statistical evaluation of cell kinetic data from DNA flow cytometry (FCM) by the EM algorithm Cytometry 1989 10 695 705 2582959 10.1002/cyto.990100605

[B24] Schutte B Reynders MM Bosman FT Blijham GH Flow cytometric determination of DNA ploidy level in nuclei isolated from paraffin-embedded tissue Cytometry 1985 6 26 30 3967551 10.1002/cyto.990060106

[B25] Shia J Immunohistochemistry versus microsatellite instability testing for screening colorectal cancer patients at risk for hereditary nonpolyposis colorectal cancer syndrome. Part I. The utility of immunohistochemistry J Mol Diagn 2008 10 293 300 18556767 10.2353/jmoldx.2008.080031 PMC2438196

[B26] Chapusot C Martin L Bouvier AM Bonithon-Kopp C Ecarnot-Laubriet A Rageot D Ponnelle T Laurent Puig P Faivre J Piard F Microsatellite instability and intratumoural heterogeneity in 100 right-sided sporadic colon carcinomas Br J Cancer 2002 87 400 404 12177776 10.1038/sj.bjc.6600474 PMC2376141

[B27] Watson N Grieu F Morris M Harvey J Stewart C Schofield L Goldblatt J Iacopetta B Heterogeneous staining for mismatch repair proteins during population-based prescreening for hereditary nonpolyposis colorectal cancer J Mol Diagn 2007 9 472 478 17652638 10.2353/jmoldx.2007.060162 PMC1975100

[B28] Nodin B Johannesson H Wangefjord S O’Connor DP Lindquist KE Uhlen M Jirstrom K Eberhard J Molecular correlates and prognostic significance of SATB1 expression in colorectal cancer Diagn Pathol 2012 7 115 22935204 10.1186/1746-1596-7-115 PMC3523011

[B29] Wangefjord S Brandstedt J Lindquist KE Nodin B Jirstrom K Eberhard J Associations of beta-catenin alterations and MSI screening status with expression of key cell cycle regulating proteins and survival from colorectal cancer Diagn Pathol 2013 8 10 23337059 10.1186/1746-1596-8-10 PMC3599130

[B30] Fadhil W Ilyas M Immunostaining for mismatch repair (MMR) protein expression in colorectal cancer is better and easier to interpret when performed on diagnostic biopsies Histopathology 2012 60 653 655 22260350 10.1111/j.1365-2559.2011.04021.x

[B31] Kumarasinghe AP de Boer B Bateman AC Kumarasinghe MP DNA mismatch repair enzyme immunohistochemistry in colorectal cancer: a comparison of biopsy and resection material Pathology 2010 42 414 420 20632816 10.3109/00313025.2010.493862

[B32] Shia J Stadler Z Weiser MR Rentz M Gonen M Tang LH Vakiani E Katabi N Xiong X Markowitz AJ Shike M Guillem J Klimstra DS Immunohistochemical staining for DNA mismatch repair proteins in intestinal tract carcinoma: how reliable are biopsy samples? Am J Surg Pathol 2011 35 447 454 21297438 10.1097/PAS.0b013e31820a091d

[B33] Warrier SK Trainer AH Lynch AC Mitchell C Hiscock R Sawyer S Boussioutas A Heriot AG Preoperative diagnosis of Lynch syndrome with DNA mismatch repair immunohistochemistry on a diagnostic biopsy Dis Colon Rectum 2011 54 1480 1487 22067175 10.1097/DCR.0b013e318231db1f

